# Supplementary material for: Iterative improvement in the automatic modular design of robot swarms
Source: PeerJ Comput Sci. 2020 Dec 7;6:e322. doi: 10.7717/peerj-cs.322 (PMC7924708; doi:10.7717/peerj-cs.322)
Supplement: Supplemental Information 3 [file peerj-cs-06-322-s003.zip › argos3/doc/api/standalone/a00574_source.html]

ARGoS: plugins/simulator/entities/led\_equipped\_entity.cpp Source File


- Main Page
- Related Pages
- Namespaces
- Classes
- Files

- File List
- File Members

# plugins/simulator/entities/led\_equipped\_entity.cpp

Go to the documentation of this file.

```
00001 
00007 #include "led_equipped_entity.h"
00008 #include <argos3/core/simulator/simulator.h>
00009 #include <argos3/core/simulator/space/space.h>
00010 #include <argos3/plugins/simulator/media/led_medium.h>
00011 
00012 namespace argos {
00013 
00014    /****************************************/
00015    /****************************************/
00016 
00017    CLEDEquippedEntity::SActuator::SActuator(CLEDEntity& c_led,
00018                                             const CVector3& c_offset,
00019                                             SAnchor& s_anchor) :
00020       LED(c_led),
00021       Offset(c_offset),
00022       Anchor(s_anchor) {}
00023 
00024    /****************************************/
00025    /****************************************/
00026 
00027    CLEDEquippedEntity::CLEDEquippedEntity(CComposableEntity* pc_parent) :
00028       CComposableEntity(pc_parent) {
00029       Disable();
00030    }
00031 
00032    /****************************************/
00033    /****************************************/
00034 
00035    CLEDEquippedEntity::CLEDEquippedEntity(CComposableEntity* pc_parent,
00036                                           const std::string& str_id) :
00037       CComposableEntity(pc_parent, str_id) {
00038       Disable();
00039    }
00040 
00041    /****************************************/
00042    /****************************************/
00043 
00044    CLEDEquippedEntity::~CLEDEquippedEntity() {
00045       while(! m_tLEDs.empty()) {
00046          delete m_tLEDs.back();
00047          m_tLEDs.pop_back();
00048       }
00049    }
00050 
00051    /****************************************/
00052    /****************************************/
00053 
00054    void CLEDEquippedEntity::Init(TConfigurationNode& t_tree) {
00055       try {
00056          /* Init parent */
00057          CComposableEntity::Init(t_tree);
00058          /* Go through the led entries */
00059          CVector3 cPosition;
00060          CColor cColor;
00061          TConfigurationNodeIterator itLED("led");
00062          for(itLED = itLED.begin(&t_tree);
00063              itLED != itLED.end();
00064              ++itLED) {
00065             /* Initialise the LED using the XML */
00066             CLEDEntity* pcLED = new CLEDEntity(this);
00067             pcLED->Init(*itLED);
00068             /* Parse the offset */
00069             CVector3 cOffset;
00070             GetNodeAttribute(*itLED, "offset", cOffset);
00071             /* Parse and look up the anchor */
00072             std::string strAnchorId;
00073             GetNodeAttribute(*itLED, "anchor", strAnchorId);
00074             /*
00075              * NOTE: here we get a reference to the embodied entity
00076              * This line works under the assumption that:
00077              * 1. the LEDEquippedEntity has a parent;
00078              * 2. the parent has a child whose id is "body"
00079              * 3. the "body" is an embodied entity
00080              * If any of the above is false, this line will bomb out.
00081              */
00082             CEmbodiedEntity& cBody = GetParent().GetComponent<CEmbodiedEntity>("body");
00083             /* Add the LED to this container */
00084             m_tLEDs.push_back(new SActuator(*pcLED, cOffset, cBody.GetAnchor(strAnchorId)));
00085             AddComponent(*pcLED);
00086          }
00087          UpdateComponents();
00088       }
00089       catch(CARGoSException& ex) {
00090          THROW_ARGOSEXCEPTION_NESTED("Failed to initialize LED equipped entity \"" << GetId() << "\".", ex);
00091       }
00092    }
00093 
00094    /****************************************/
00095    /****************************************/
00096 
00097    void CLEDEquippedEntity::Reset() {
00098       for(SActuator::TList::iterator it = m_tLEDs.begin();
00099           it != m_tLEDs.end();
00100           ++it) {
00101          (*it)->LED.Reset();
00102       }
00103    }
00104 
00105    /****************************************/
00106    /****************************************/
00107 
00108    void CLEDEquippedEntity::Enable() {
00109       CEntity::Enable();
00110       for(size_t i = 0; i < m_tLEDs.size(); ++i) {
00111          m_tLEDs[i]->Anchor.Enable();
00112       }
00113    }
00114 
00115    /****************************************/
00116    /****************************************/
00117 
00118    void CLEDEquippedEntity::Disable() {
00119       CEntity::Disable();
00120       for(size_t i = 0; i < m_tLEDs.size(); ++i) {
00121          m_tLEDs[i]->Anchor.Disable();
00122       }
00123    }
00124 
00125    /****************************************/
00126    /****************************************/
00127 
00128    void CLEDEquippedEntity::AddLED(const CVector3& c_offset,
00129                                    SAnchor& s_anchor,
00130                                    const CColor& c_color) {
00131       CLEDEntity* pcLED =
00132          new CLEDEntity(
00133             this,
00134             std::string("led_") + ToString(m_tLEDs.size()),
00135             c_offset,
00136             c_color);
00137       m_tLEDs.push_back(new SActuator(*pcLED, c_offset, s_anchor));
00138       AddComponent(*pcLED);
00139    }
00140 
00141    /****************************************/
00142    /****************************************/
00143 
00144    void CLEDEquippedEntity::AddLEDRing(const CVector3& c_center,
00145                                        Real f_radius,
00146                                        const CRadians& c_start_angle,
00147                                        UInt32 un_num_leds,
00148                                        SAnchor& s_anchor,
00149                                        const CColor& c_color) {
00150       CRadians cLEDSpacing = CRadians::TWO_PI / un_num_leds;
00151       CRadians cAngle;
00152       CVector3 cOffset;
00153       for(UInt32 i = 0; i < un_num_leds; ++i) {
00154          cAngle = c_start_angle + i * cLEDSpacing;
00155          cAngle.SignedNormalize();
00156          cOffset.Set(f_radius, 0.0f, 0.0f);
00157          cOffset.RotateZ(cAngle);
00158          cOffset += c_center;
00159          AddLED(cOffset, s_anchor, c_color);
00160       }
00161    }
00162 
00163    /****************************************/
00164    /****************************************/
00165 
00166    CLEDEntity& CLEDEquippedEntity::GetLED(UInt32 un_index) {
00167       ARGOS_ASSERT(un_index < m_tLEDs.size(),
00168                    "CLEDEquippedEntity::GetLED(), id=\"" <<
00169                    GetId() <<
00170                    "\": index out of bounds: un_index = " <<
00171                    un_index <<
00172                    ", m_tLEDs.size() = " <<
00173                    m_tLEDs.size());
00174       return m_tLEDs[un_index]->LED;
00175    }
00176 
00177    /****************************************/
00178    /****************************************/
00179 
00180    void CLEDEquippedEntity::SetLEDOffset(UInt32 un_index,
00181                                          const CVector3& c_offset) {
00182       ARGOS_ASSERT(un_index < m_tLEDs.size(),
00183                    "CLEDEquippedEntity::SetLEDPosition(), id=\"" <<
00184                    GetId() <<
00185                    "\": index out of bounds: un_index = " <<
00186                    un_index <<
00187                    ", m_tLEDs.size() = " <<
00188                    m_tLEDs.size());
00189       m_tLEDs[un_index]->Offset = c_offset;
00190    }
00191 
00192    /****************************************/
00193    /****************************************/
00194 
00195    void CLEDEquippedEntity::SetLEDColor(UInt32 un_index,
00196                                         const CColor& c_color) {
00197       ARGOS_ASSERT(un_index < m_tLEDs.size(),
00198                    "CLEDEquippedEntity::SetLEDColor(), id=\"" <<
00199                    GetId() <<
00200                    "\": index out of bounds: un_index = " <<
00201                    un_index <<
00202                    ", m_tLEDs.size() = " <<
00203                    m_tLEDs.size());
00204       m_tLEDs[un_index]->LED.SetColor(c_color);
00205    }
00206 
00207    /****************************************/
00208    /****************************************/
00209 
00210    void CLEDEquippedEntity::SetAllLEDsColors(const CColor& c_color) {
00211       for(UInt32 i = 0; i < m_tLEDs.size(); ++i) {
00212          m_tLEDs[i]->LED.SetColor(c_color);
00213       }
00214    }
00215 
00216    /****************************************/
00217    /****************************************/
00218 
00219    void CLEDEquippedEntity::SetAllLEDsColors(const std::vector<CColor>& vec_colors) {
00220       if(vec_colors.size() == m_tLEDs.size()) {
00221          for(UInt32 i = 0; i < vec_colors.size(); ++i) {
00222             m_tLEDs[i]->LED.SetColor(vec_colors[i]);
00223          }
00224       }
00225       else {
00226          THROW_ARGOSEXCEPTION(
00227             "CLEDEquippedEntity::SetAllLEDsColors(), id=\"" <<
00228             GetId() <<
00229             "\": number of LEDs (" <<
00230             m_tLEDs.size() <<
00231             ") is lower than the passed color vector size (" <<
00232             vec_colors.size() <<
00233             ")");
00234       }
00235    }
00236 
00237    /****************************************/
00238    /****************************************/
00239 
00240    void CLEDEquippedEntity::UpdateComponents() {
00241       /* LED position wrt global reference frame */
00242       CVector3 cLEDPosition;
00243       for(UInt32 i = 0; i < m_tLEDs.size(); ++i) {
00244          if(m_tLEDs[i]->LED.IsEnabled()) {
00245             cLEDPosition = m_tLEDs[i]->Offset;
00246             cLEDPosition.Rotate(m_tLEDs[i]->Anchor.Orientation);
00247             cLEDPosition += m_tLEDs[i]->Anchor.Position;
00248             m_tLEDs[i]->LED.SetPosition(cLEDPosition);
00249          }
00250       }
00251    }
00252 
00253    /****************************************/
00254    /****************************************/
00255 
00256    void CLEDEquippedEntity::AddToMedium(CLEDMedium& c_medium) {
00257       for(UInt32 i = 0; i < m_tLEDs.size(); ++i) {
00258          m_tLEDs[i]->LED.AddToMedium(c_medium);
00259       }
00260       Enable();
00261    }
00262 
00263    /****************************************/
00264    /****************************************/
00265 
00266    void CLEDEquippedEntity::RemoveFromMedium() {
00267       for(UInt32 i = 0; i < m_tLEDs.size(); ++i) {
00268          m_tLEDs[i]->LED.RemoveFromMedium();
00269       }
00270       Disable();
00271    }
00272 
00273    /****************************************/
00274    /****************************************/
00275 
00276    REGISTER_STANDARD_SPACE_OPERATIONS_ON_COMPOSABLE(CLEDEquippedEntity);
00277 
00278    /****************************************/
00279    /****************************************/
00280 
00281 }
```

---

Generated on 10 Jul 2018 for ARGoS by 
 1.6.1
